# Tropically stable novel oral lipid formulation of amphotericin B (iCo-010): biodistribution and toxicity in a mouse model

**DOI:** 10.1186/1476-511X-10-135

**Published:** 2011-08-08

**Authors:** Olena Sivak, Pavel Gershkovich, Molly Lin, Ellen K Wasan, Jinying Zhao, David Owen, John G Clement, Kishor M Wasan

**Affiliations:** 1 Faculty of Pharmaceutical Sciences, University of British Columbia, 2146 East Mall, Vancouver, BC, Canada V6T 1Z3; 2 School of Health Sciences, British Columbia Institute of Technology, 3700 Willingdon Avenue, Burnaby, BC, Canada, V5G 3H2; 3 Department of Pathology & Laboratory Medicine, University of British Columbia, 855 West 12th Avenue, Vancouver, BC, Canada, V5Z 1M9; 4 iCo Therapeutics Inc., 760-777 Hornby, Vancouver, BC, Canada V6Z 1S4

**Keywords:** Lipid-based formulation, Intestinal absorption, Nephrotoxicity, Tissue distribution, Amphotericin B

## Abstract

**Background:**

The purpose of this study was to evaluate the biodistribution and toxicity of amphotericin B (AmB) following multiple oral administrations of a novel tropically stable lipid-based formulation (iCo-010).

**Methods:**

BALB/c mice were allocated into six groups: oral iCo-010 twice daily for 5 days in the dose of 20, 10, 5 and 2.5 mg/kg; vehicle control; and intravenous boluses of Fungizone^® ^2 mg/kg once daily for 5 days. The animals were sacrificed 12 h following the last administration and blood and tissues were collected.

**Results:**

The plasma concentrations of AmB were similar to previously reported after administration of iCo-009. Somewhat lower concentrations of AmB were detected in reticulo-endothelial system in the case of iCo-010 when compared with iCo-009. The concentration in kidney was higher with iCo-010 than with iCo-009. The creatinine levels in all oral treatment groups were in a normal range as in the case of iCo-009. Administration of Fungizone^® ^resulted in elevated plasma creatinine levels. Histopathology analysis detected no GI, liver or kidney toxicity following multiple dose oral administration of iCo-010. Fungizone^® ^treatment induced necrotic changes in hepatic and kidney tissues.

**Conclusions:**

Given the tropical stability of iCo-010, near identical activity against visceral leishmaniasis and significant concentrations in target organs this formulation has a potential to become a treatment of choice in tropical developing countries.

## Findings

Amphotericin B (AmB) is a polyene antibiotic that is used in treatment of systemic fungal and parasitic infections. The major limitations of existing AmB therapies are the toxicity of the drug (mainly to kidneys) and the need for parenteral administration due to poor oral bioavailability [1]. Parenteral lipid-based formulations of AmB such as Abelcet^® ^and Ambisome^® ^significantly decreased the toxicity associated with the treatment. However, the need to administer the drug intravenously and high costs associated with these lipid-based formulations are still being serious limitations of the current treatments, especially in developing countries [2]. Experimental lipid-based parenteral formulations of AmB based on less expensive ingredients have been reported [3,4].

Oral lipid-based formulation of AmB (iCo-009) composed of monoglycerides, diglycerides and distearoylphosphatidylethanolamine polyethylene glycol 2000 [5] has been previously reported to be effective against systemic fungal infections in rats [5] and against visceral leishmaniasis in mice [6]. The biodistribution pattern and the lack of kidney, liver or gastrointestinal toxicity in mice [7] supported the efficacy data and suggested that this lipid-based formulation has a promising potential in treating fungal and parasitic infections. However, the insufficient tropical temperature stability limited the potential of iCo-009 for use in developing third-world countries with hot climate. The oral form of AmB is most needed in poor tropical countries due to spread of diseases such as leishmaniasis on one hand and the lack of appropriate infrastructure and financial struggles that limit the parenteral administration of amphotericin B on the other hand. Keeping the challenge of tropical climate stability in mind, a new oral lipid-based formulation of AmB (iCo-010) composed of monoglycerides, diglycerides, polyethylene glycol glycerides and D-alpha-tocopheryl polyethylene glycol succinate [8] has been developed and tested against visceral leishmaniasis in a murine model. The formulation showed slightly decreased activity against visceral leishmaniasis comparing to iCo-009 (but still about 99% inhibition at the highest dose of 10 mg/kg twice daily (BID) for 5 days) and very good stability at tropical temperatures [8].

The purpose of the current work was to evaluate the tissue distribution and toxicity of AmB following multiple dose oral administration of a novel tropically stable lipid-based formulation (iCo-010). In addition to tissues that were examined in our previous work with iCo-009 [7,9], in this study we have added additional organs (skin, muscle and adipose tissue). An additional novel aspect of this work was that tissue concentrations of AmB following multiple intravenous (IV) bolus administrations of Fungizone^® ^were also measured and compared to the concentrations obtained after multiple oral dosing, a head to head comparison that was not done in our previous works with multiple administrations of older formulations.

## Methods

The lipid-based oral formulation of AmB (iCo-10) was prepared as recently reported [8]. AmB powder and 1-Amino-4-nitronaphtalene (internal standard) were purchased from Sigma-Aldrich (St. Louis, MO, USA). Fungizone^® ^(AmB micellular dispersion, Bristol-Myers Squibb, Montreal, Canada), was purchased from Vancouver General Hospital pharmacy. All other chemicals were of analytical reagent grade, and solvents were of HPLC grade.

The use of animals for this study was approved by the University of British Columbia's Animal Care Committee and all experimental protocols conform to the Canadian Council on Animal Care guidelines. BALB/c female mice with a body weight of 18-20 g (Charles River Laboratories, Wilmington, MA, USA) were used in this work. Following a one week acclimatization period animals were allocated into the following groups: oral gavages of iCo-010 twice daily for 5 days in the dose of 20 mg/kg (n = 7), 10 mg/kg (n = 6), 5 mg/kg (n = 6) and 2.5 mg/kg (n = 6); oral gavages of vehicle control of iCo-010 (n = 5); and intravenous (IV) boluses of Fungizone^® ^once daily for 5 days in the dose that was shown to be nephrotoxic in a previously reported study (2 mg/kg, n = 16) [3]. The animals were sacrificed 12 h following the last administration of AmB, blood and tissues were collected for drug analysis and histopathological evaluation.

Plasma and tissue samples were analyzed for concentrations of AmB by HPLC as previously reported [7]. Plasma samples were analyzed for creatinine concentrations using previously published validated HPLC method [10].

Right kidney, a portion of jejunum and a median lobe of liver were removed and fixated in 10% neutral buffered formalin for histopathological analysis.

## Results and Discussion

**Figure 1:**
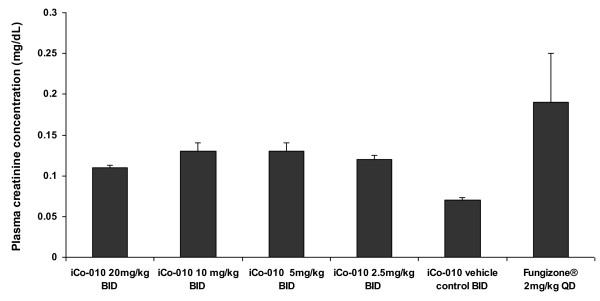
**Plasma creatinine concentrations (Mean ± SEM; mg/dL) in mice 12 hours following the completion of twice-daily × 5 days treatment course of different doses of iCo-010 (n = 5-6) or 12 hours following the completion of once daily × 5 days IV treatment course of Fungizone^® ^2 mg/kg (n = 6)**. Ten (out of 16) animals were lost at different times during the dosing of Fungizone^® ^due to acute toxicity associated with this dose.

**Figure 2:**
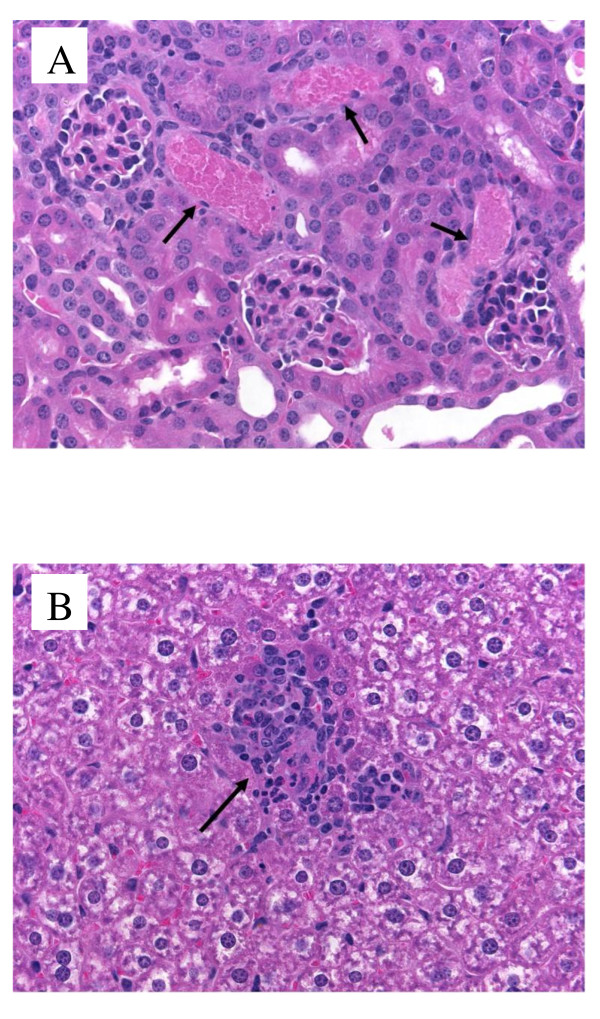
**Panel A - representative kidney histopathology of a mouse from the 2 mg/kg iv Fungizone^® ^group (arrows show tubules with necrotic debris)**. Panel B - representative liver histopathology of a mouse from the 2 mg/kg iv Fungizone^® ^group (arrow show a focal collection of necrotic hepatocytes). The histopathology analysis of liver and kidney tissue of animals in all oral treatment groups and of jejunum tissue in all treatment groups showed normal morphology pattern (data not shown).

The concentrations of AmB in plasma and multiple organs 12 hours following the administration of the last dose of iCo-010 or Fungizone^® ^are shown in Table 1. The plasma concentrations following the administration of iCo-010 are similar to previously reported after multiple dose administration of another lipid-based formulation, iCo-009 [7]. However, there are considerable differences in tissue distribution pattern. Lower concentrations of AmB are detected in reticulo-endothelial system organs in the case of iCo-010 than has been reported previously with iCo-009 [7]. This is consistent with slightly lower activity of iCo-010 in a murine model of visceral leishmaniasis [6,8]. However, the marginal reduction in activity comes with increased tropical stability of iCo-010 [8], which is an important advantage given the hot climate in areas where the leishmaniasis is spread and the limited refrigeration capabilities in these regions. Interestingly, concentrations in kidney tissues obtained in this study following the administration of iCo-010 (Table 1) were significantly higher than after administration of iCo-009 [5]. Although differences in the rate and extent of intestinal absorption between iCo-009 and iCo-010 cannot be ruled out at this moment, opposite direction of changes in reticulo-endothelial system and in kidney and similar concentrations in plasma and brain suggest that dissimilarity in concentrations of AmB in tissues after administration of two lipid-based formulations is related mostly to differences in post-absorptive distribution. When concentrations of AmB in tissues are compared to those obtained in plasma after the oral administration of iCo-010, it can be seen that AmB is preferentially accumulated in liver, spleen, lung and adipose tissues (Table 1). The levels in heart, brain, skin and skeletal muscle are not statistically significantly different from levels observed in plasma. In the Fungizone^® ^group a similar pattern of preferential accumulation in reticulo-endothelial and adipose tissues can be observed. However, due to high variability in this group only AmB concentrations in liver and spleen tissues were found to be statistically different from levels in plasma. The relatively high concentration obtained in adipose tissues (in iCo-010 and in Fungizone^® ^groups) suggests that adipose tissue can create an important depot compartment of AmB that was not previously taken into consideration in pharmacokinetic calculations and modeling of AmB [11]. The relatively low levels observed in muscle tissues can still be significant in terms of total amount of the drug residing in these tissues due to high mass of muscle tissues in the body. The levels obtained in skin tissue after administration of iCo-10 or Fungizone^® ^were significant, but not different from plasma. The lack of preferential accumulation in skin suggests that longer treatment regimens would be needed for treatment of cutaneous or mucocutaneous forms of leishmaniasis than for visceral form of the disease. The creatinine levels in all oral treatment groups (Figure 1) were in a normal range and similar to previously reported values after administration of iCo-009 [5]. Administration of IV boluses of Fungizone^® ^2 mg/kg resulted in acute toxicity (ten animals (out of 16) were lost within minutes following one of the dosings). The plasma creatinine levels in six animals in this group that survived all five administrations reached mild renal toxicity levels (Figure 1). As with iCo-009 [5] histopathology analysis detected no intestine, liver or kidney toxicity in this study following multiple dose oral administration of iCo-010, including at the highest dose (20 mg/kg) (data not shown). On the other hand Fungizone^® ^treatment induced necrotic changes in hepatic and kidney tissues identical to those reported in our previous study [5] (Figure 2A and 2B).

**Table 1:** Tissue concentration of amphotericin B (Mean ± SEM; ng/g) in mice 12 hours following the completion of twice-daily × 5 days treatment course of different doses of oral tropically stable formulation of AmB (iCo-010) or 12 hours following the completion of once daily × 5 days IV treatment course of Fungizone^® ^2 mg/kg

	iCo-010 20 mg/kg BID for 5 days, n = 7^a^	iCo-010 10 mg/kg BID for 5 days, n = 6^b^	**iCo-010 5 mg/kg BID for 5 days, n = 6**^c^	iCo-010 2.5 mg/kg BID for 5 days, n = 6^b^	Fungizone^®^ **2 mg/kg QD for 5 days, n = 6**^d^
Plasma	538 ± 27	418 ± 48	232 ± 22	172 ± 10	791 ± 90

Liver	3494 ± 287	2543 ± 510	836 ± 97	446 ± 44	49794 ± 6993

Spleen	1939 ± 87	1407 ± 120	916 ± 121	342 ± 20	27008 ± 2400

Lung	3179 ± 312	2014 ± 185	1168 ± 307	408 ± 47	7533 ± 1299

Kidney	3685 ± 334	2268 ± 220	813 ± 89	495 ± 31	9100 ± 1140

Heart	366 ± 31	338 ± 41	156 ± 10	84 ± 8	1139 ± 243

Brain	169 ± 6	157 ± 3	112 ± 10	59 ± 5	234 ± 23

Skin	393 ± 45	165 ± 19	116 ± 20	BLQ	420 ± 269

Muscle	26 ± 16	24 ± 8	BLQ	BLQ	468 ± 148

Visceral Fat	2114 ± 512	904 ± 220	471 ± 112	BLQ	3324 ± 654

^a^AmB concentration in plasma is statistically significantly different from concentration in liver (p < 0.01), spleen (p < 0.01), kidney (p < 0.01), lung (p < 0.01) and fat tissue (p < 0.01) within the same treatment group (One-way ANOVA followed by Dunnett Multiple Comparisons Test).

^b^AmB concentration in plasma is statistically significantly different from concentration in liver (p < 0.01), spleen (p < 0.01), kidney (p < 0.01) and lung (p < 0.01) within the same treatment group (One-way ANOVA followed by Dunnett Multiple Comparisons Test).

^c^AmB concentration in plasma is statistically significantly different from concentration in liver (p < 0.01), spleen (p < 0.01), kidney (p < 0.05) and lung (p < 0.01) within the same treatment group (One-way ANOVA followed by Dunnett Multiple Comparisons Test).

^d^AmB concentration in plasma is statistically significantly different from concentration in liver (p < 0.01) and spleen (p < 0.01) within the same treatment group (One-way ANOVA followed by Dunnett Multiple Comparisons Test).

In conclusion, in the current work the biodistrbution and toxicity of a novel tropically stable oral lipid-based formulation of AmB (iCo-010) was assessed in a mouse model. Although the levels in reticulo-endothelial system (target organs in treatment of visceral leishmaniasis) were somewhat lower than with previously described (tropically unstable) oral formulation of AmB (iCo-009) [7], the concentrations were still well above the IC_50 _(reported to be about 50-100 ng/ml [12,13]) for leishmania organism at all tested doses. These data support the previously reported near identical activity of iCo-009 and iCo-010 in a murine model of visceral leishmaniasis [6,8]. The concentrations in kidney tissue following multiple dose administration of iCo-010 exceeded those reported with iCo-009. Plasma creatinine measurement and kidney, liver and jejunum histopathology analysis have not detected any toxicity signs following the administration of iCo-010.

Taking into consideration the tropical stability of iCo-010, near identical (to iCo-009) activity against visceral leishmaniasis and significant concentrations in target organs this formulation has a potential to become the treatment of choice in developing countries where the oral treatment of visceral leishmaniasis is most needed.

## List of abbreviations

AmB: amphotericin B; IV: intravenous; BID: twice daily; QD: once daily

## Competing interests

J. G. C. is an employee/co-founder/shareholder and director of iCo Therapeutics Inc. All other authors declare no conflict of interests.

## Authors' contributions

OS carried out the majority of animal experiments, analysis of the drug and participated in the design of the study; PG carried out animal experiments, analysis of the drug, participated in the design of the study and wrote the manuscript; ML participated in animal experiments and analysis of the drug; EKW developed the lipid formulation of AmB (iCo-010); JZ prepared the iCo-010 formulation; DO carried out histopathology analysis; JGC participated in the design of the study and in the discussion of the results; KMW coordinated the work of the research team, participated in the design of the study and in the analysis of the results. All the authors read and approved the final manuscript.
